# *N*-Butyladamantane-1-carboxamide

**DOI:** 10.1107/S1600536809036897

**Published:** 2009-09-19

**Authors:** Weiwei SiMa

**Affiliations:** a Ordered Matter Science Research Center, College of Chemistry and Chemical Engineering, Southeast University, Nanjing 210096, People’s Republic of China

## Abstract

In the crystal of the title compound, C_15_H_25_NO, the mol­ecules are linked into chains propagating in [001] by inter­molecular N—H⋯O hydrogen bonds.

## Related literature

For a related structure, see: SiMa (2009 ▶). For further synthetic details, see: Tadashi & Sasaki (1969 ▶).
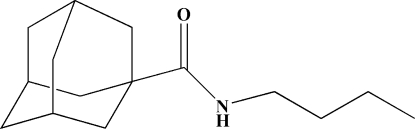


## Experimental

### 

#### Crystal data

C_15_H_25_NO
*M* *_r_* = 235.36
Monoclinic, 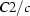

*a* = 32.257 (7) Å
*b* = 9.4353 (19) Å
*c* = 9.5328 (19) Å
β = 101.69 (3)°
*V* = 2841.1 (10) Å^3^
*Z* = 8
Mo *K*α radiation
μ = 0.07 mm^−1^
*T* = 298 K
0.20 × 0.20 × 0.20 mm

#### Data collection

Rigaku SCXmini diffractometer
Absorption correction: none
14458 measured reflections
3260 independent reflections
1544 reflections with *I* > 2σ(*I*)
*R*_int_ = 0.077

#### Refinement

*R*[*F* ^2^ > 2σ(*F* ^2^)] = 0.086
*wR*(*F* ^2^) = 0.246
*S* = 1.04
3260 reflections
155 parameters
H-atom parameters constrained
Δρ_max_ = 0.36 e Å^−3^
Δρ_min_ = −0.32 e Å^−3^

### 

Data collection: *CrystalClear* (Rigaku, 2005 ▶); cell refinement: *CrystalClear*; data reduction: *CrystalClear*; program(s) used to solve structure: *SHELXS97* (Sheldrick, 2008 ▶); program(s) used to refine structure: *SHELXL97* (Sheldrick, 2008 ▶); molecular graphics: *SHELXTL* (Sheldrick, 2008 ▶); software used to prepare material for publication: *SHELXL97*.

## Supplementary Material

Crystal structure: contains datablocks I, New_Global_Publ_Block. DOI: 10.1107/S1600536809036897/hb5069sup1.cif

Structure factors: contains datablocks I. DOI: 10.1107/S1600536809036897/hb5069Isup2.hkl

Additional supplementary materials:  crystallographic information; 3D view; checkCIF report

## Figures and Tables

**Table 1:** Hydrogen-bond geometry (Å, °)

*D*—H⋯*A*	*D*—H	H⋯*A*	*D*⋯*A*	*D*—H⋯*A*
N1—H1*C*⋯O1^i^	0.99	1.96	2.896 (3)	158

Symmetry code: (i) 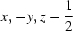.

**Fig. 1.**
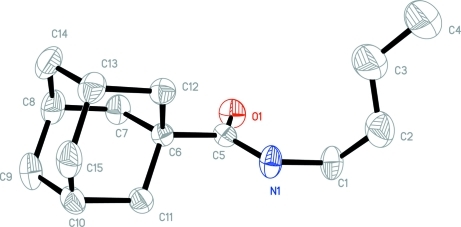
The molecular structure of (I). Displacement ellipsoids are drawn at the 30% probability level, and all H atoms have been omitted for clarity.

## supplementary crystallographic information

### Experimental

A solution of freshly prepared 1-adamantane carbonyl chloride (1 mmol, prepared by refluxing 1-adamantane carboxylic acid with 3*M* excess of SOCl_2_) in dry CH_2_Cl_2_ was added dropwise to a well stirred and ice-cooled solution of butanamine (1 mmol) and triethylamine (2 mmol) in the same solvent. After 24 h of stirring at room temperature, the solvents were removed *in vacuo* and the residue was recrystallized from methanol. Colourless prisms of (I) were obtained in 80% yield (Tadashi Sasaki *et al.*, 1969).

### Refinement

Positional parameters of all the H atoms were calculated geometrically and were allowed to ride on the N or C atoms to which they are bonded, with *U*_iso_(H) = 1.2*U*_eq_(N,C) or 1.5*U*_eq_(methyl C).

### Crystal data

**Table:**

C_15_H_25_NO	*F*(000) = 1040
*M**_r_* = 235.36	*D*_x_ = 1.100 Mg m^−^^3^
Monoclinic, *C*2/*c*	Mo *K*α radiation, λ = 0.71073 Å
Hall symbol: -C 2yc	Cell parameters from 4730 reflections
*a* = 32.257 (7) Å	θ = 3.0–27.8°
*b* = 9.4353 (19) Å	µ = 0.07 mm^−^^1^
*c* = 9.5328 (19) Å	*T* = 298 K
β = 101.69 (3)°	Prism, colourless
*V* = 2841.1 (10) Å^3^	0.20 × 0.20 × 0.20 mm
*Z* = 8	

### Data collection

**Table:**

Rigaku SCXmini diffractometer	1544 reflections with *I* > 2σ(*I*)
Radiation source: fine-focus sealed tube	*R*_int_ = 0.077
graphite	θ_max_ = 27.5°, θ_min_ = 3.0°
Detector resolution: 13.6612 pixels mm^-1^	*h* = −41→41
ω scans	*k* = −12→12
14458 measured reflections	*l* = −12→12
3260 independent reflections	

### Refinement

**Table:**

Refinement on *F*^2^	Primary atom site location: structure-invariant direct methods
Least-squares matrix: full	Secondary atom site location: difference Fourier map
*R*[*F*^2^ > 2σ(*F*^2^)] = 0.086	Hydrogen site location: inferred from neighbouring sites
*wR*(*F*^2^) = 0.246	H-atom parameters constrained
*S* = 1.04	*w* = 1/[σ^2^(*F*_o_^2^) + (0.0977*P*)^2^ + 2.030*P*] where *P* = (*F*_o_^2^ + 2*F*_c_^2^)/3
3260 reflections	(Δ/σ)_max_ < 0.001
155 parameters	Δρ_max_ = 0.36 e Å^−^^3^
0 restraints	Δρ_min_ = −0.32 e Å^−^^3^

### Special details

**Table:**

Geometry. All e.s.d.'s (except the e.s.d. in the dihedral angle between two l.s. planes) are estimated using the full covariance matrix. The cell e.s.d.'s are taken into account individually in the estimation of e.s.d.'s in distances, angles and torsion angles; correlations between e.s.d.'s in cell parameters are only used when they are defined by crystal symmetry. An approximate (isotropic) treatment of cell e.s.d.'s is used for estimating e.s.d.'s involving l.s. planes.
Refinement. Refinement of *F*^2^ against ALL reflections. The weighted *R*-factor *wR* and goodness of fit *S* are based on *F*^2^, conventional *R*-factors *R* are based on *F*, with *F* set to zero for negative *F*^2^. The threshold expression of *F*^2^ > σ(*F*^2^) is used only for calculating *R*-factors(gt) *etc*. and is not relevant to the choice of reflections for refinement. *R*-factors based on *F*^2^ are statistically about twice as large as those based on *F*, and *R*- factors based on ALL data will be even larger.

### Fractional atomic coordinates and isotropic or equivalent isotropic displacement parameters (Å^2^)

**Table:**

	*x*	*y*	*z*	*U*_iso_*/*U*_eq_	
C1	0.30657 (10)	−0.1173 (4)	0.4805 (4)	0.0671 (10)	
H1A	0.3025	−0.1014	0.5774	0.080*	
H1B	0.3146	−0.2157	0.4733	0.080*	
C2	0.26562 (13)	−0.0911 (5)	0.3781 (5)	0.0935 (13)	
H2A	0.2702	−0.1038	0.2813	0.112*	
H2B	0.2453	−0.1622	0.3941	0.112*	
C3	0.24721 (15)	0.0484 (6)	0.3883 (7)	0.143 (2)	
H3A	0.2643	0.1164	0.3488	0.172*	
H3B	0.2502	0.0705	0.4892	0.172*	
C4	0.20314 (15)	0.0729 (6)	0.3200 (7)	0.152 (2)	
H4A	0.1997	0.0609	0.2183	0.229*	
H4B	0.1952	0.1677	0.3403	0.229*	
H4C	0.1854	0.0065	0.3564	0.229*	
C5	0.36027 (8)	0.0693 (3)	0.5470 (3)	0.0431 (7)	
C6	0.39355 (8)	0.1604 (3)	0.4964 (3)	0.0411 (7)	
C7	0.41628 (10)	0.2560 (4)	0.6189 (3)	0.0615 (9)	
H7A	0.3957	0.3154	0.6524	0.074*	
H7B	0.4303	0.1979	0.6984	0.074*	
C8	0.44885 (11)	0.3491 (4)	0.5667 (4)	0.0715 (10)	
H8	0.4630	0.4095	0.6456	0.086*	
C9	0.48163 (10)	0.2564 (4)	0.5166 (4)	0.0795 (11)	
H9A	0.5025	0.3155	0.4844	0.095*	
H9B	0.4961	0.1981	0.5952	0.095*	
C10	0.45958 (10)	0.1630 (4)	0.3948 (4)	0.0614 (9)	
H10	0.4806	0.1031	0.3624	0.074*	
C11	0.42699 (9)	0.0688 (3)	0.4458 (3)	0.0525 (8)	
H11A	0.4411	0.0091	0.5238	0.063*	
H11B	0.4134	0.0078	0.3680	0.063*	
C12	0.37184 (9)	0.2551 (3)	0.3720 (3)	0.0513 (8)	
H12A	0.3576	0.1965	0.2932	0.062*	
H12B	0.3507	0.3140	0.4030	0.062*	
C13	0.40482 (11)	0.3492 (3)	0.3218 (3)	0.0619 (9)	
H13	0.3907	0.4089	0.2422	0.074*	
C14	0.42639 (12)	0.4422 (4)	0.4441 (4)	0.0773 (11)	
H14A	0.4056	0.5012	0.4765	0.093*	
H14B	0.4467	0.5036	0.4121	0.093*	
C15	0.43746 (11)	0.2543 (4)	0.2710 (3)	0.0674 (10)	
H15A	0.4234	0.1945	0.1929	0.081*	
H15B	0.4581	0.3125	0.2364	0.081*	
N1	0.34081 (7)	−0.0273 (3)	0.4547 (2)	0.0582 (7)	
H1C	0.3501	−0.0278	0.3622	0.070*	
O1	0.35154 (7)	0.0859 (2)	0.6660 (2)	0.0619 (7)	

### Atomic displacement parameters (Å^2^)

**Table:**

	*U*^11^	*U*^22^	*U*^33^	*U*^12^	*U*^13^	*U*^23^
C1	0.062 (2)	0.075 (2)	0.065 (2)	−0.0255 (18)	0.0153 (18)	0.0110 (18)
C2	0.074 (3)	0.087 (3)	0.123 (4)	−0.018 (2)	0.028 (3)	0.022 (3)
C3	0.086 (3)	0.105 (4)	0.244 (7)	0.001 (3)	0.044 (4)	0.024 (4)
C4	0.085 (4)	0.126 (5)	0.245 (8)	0.003 (3)	0.030 (4)	0.008 (5)
C5	0.0435 (16)	0.0514 (18)	0.0365 (14)	0.0034 (13)	0.0128 (13)	0.0079 (13)
C6	0.0425 (15)	0.0466 (16)	0.0357 (14)	−0.0005 (12)	0.0111 (12)	−0.0016 (12)
C7	0.069 (2)	0.072 (2)	0.0451 (17)	−0.0157 (18)	0.0151 (15)	−0.0170 (15)
C8	0.076 (2)	0.078 (2)	0.061 (2)	−0.032 (2)	0.0136 (19)	−0.0210 (19)
C9	0.055 (2)	0.098 (3)	0.085 (3)	−0.029 (2)	0.0109 (19)	−0.003 (2)
C10	0.0479 (17)	0.071 (2)	0.072 (2)	−0.0038 (16)	0.0278 (17)	−0.0084 (18)
C11	0.0473 (16)	0.0553 (19)	0.0565 (18)	0.0001 (14)	0.0142 (14)	0.0015 (14)
C12	0.0528 (17)	0.0508 (18)	0.0497 (17)	−0.0002 (14)	0.0090 (14)	0.0037 (14)
C13	0.070 (2)	0.056 (2)	0.060 (2)	−0.0064 (16)	0.0150 (17)	0.0139 (16)
C14	0.093 (3)	0.050 (2)	0.094 (3)	−0.0227 (19)	0.032 (2)	−0.0086 (19)
C15	0.070 (2)	0.081 (2)	0.059 (2)	−0.0260 (19)	0.0307 (17)	−0.0057 (18)
N1	0.0609 (16)	0.0749 (18)	0.0441 (13)	−0.0258 (14)	0.0234 (12)	−0.0055 (13)
O1	0.0715 (15)	0.0796 (16)	0.0407 (11)	−0.0063 (12)	0.0263 (11)	0.0002 (11)

### Geometric parameters (Å, °)

**Table:**

C1—N1	1.453 (4)	C8—C9	1.522 (5)
C1—C2	1.496 (5)	C8—C14	1.523 (5)
C1—H1A	0.9700	C8—H8	0.9800
C1—H1B	0.9700	C9—C10	1.516 (5)
C2—C3	1.456 (6)	C9—H9A	0.9700
C2—H2A	0.9700	C9—H9B	0.9700
C2—H2B	0.9700	C10—C15	1.517 (5)
C3—C4	1.457 (6)	C10—C11	1.529 (4)
C3—H3A	0.9700	C10—H10	0.9800
C3—H3B	0.9700	C11—H11A	0.9700
C4—H4A	0.9600	C11—H11B	0.9700
C4—H4B	0.9600	C12—C13	1.535 (4)
C4—H4C	0.9600	C12—H12A	0.9700
C5—O1	1.232 (3)	C12—H12B	0.9700
C5—N1	1.333 (3)	C13—C14	1.512 (4)
C5—C6	1.527 (4)	C13—C15	1.533 (5)
C6—C11	1.535 (4)	C13—H13	0.9800
C6—C12	1.536 (4)	C14—H14A	0.9700
C6—C7	1.539 (4)	C14—H14B	0.9700
C7—C8	1.528 (4)	C15—H15A	0.9700
C7—H7A	0.9700	C15—H15B	0.9700
C7—H7B	0.9700	N1—H1C	0.9861
			
N1—C1—C2	113.2 (3)	C10—C9—C8	109.1 (3)
N1—C1—H1A	108.9	C10—C9—H9A	109.9
C2—C1—H1A	108.9	C8—C9—H9A	109.9
N1—C1—H1B	108.9	C10—C9—H9B	109.9
C2—C1—H1B	108.9	C8—C9—H9B	109.9
H1A—C1—H1B	107.8	H9A—C9—H9B	108.3
C3—C2—C1	115.1 (4)	C9—C10—C15	109.8 (3)
C3—C2—H2A	108.5	C9—C10—C11	109.9 (3)
C1—C2—H2A	108.5	C15—C10—C11	109.4 (3)
C3—C2—H2B	108.5	C9—C10—H10	109.2
C1—C2—H2B	108.5	C15—C10—H10	109.2
H2A—C2—H2B	107.5	C11—C10—H10	109.2
C2—C3—C4	119.2 (5)	C10—C11—C6	110.2 (2)
C2—C3—H3A	107.5	C10—C11—H11A	109.6
C4—C3—H3A	107.5	C6—C11—H11A	109.6
C2—C3—H3B	107.5	C10—C11—H11B	109.6
C4—C3—H3B	107.5	C6—C11—H11B	109.6
H3A—C3—H3B	107.0	H11A—C11—H11B	108.1
C3—C4—H4A	109.5	C13—C12—C6	110.0 (2)
C3—C4—H4B	109.5	C13—C12—H12A	109.7
H4A—C4—H4B	109.5	C6—C12—H12A	109.7
C3—C4—H4C	109.5	C13—C12—H12B	109.7
H4A—C4—H4C	109.5	C6—C12—H12B	109.7
H4B—C4—H4C	109.5	H12A—C12—H12B	108.2
O1—C5—N1	122.0 (2)	C14—C13—C15	110.1 (3)
O1—C5—C6	121.7 (3)	C14—C13—C12	109.6 (3)
N1—C5—C6	116.3 (2)	C15—C13—C12	108.9 (3)
C5—C6—C11	111.5 (2)	C14—C13—H13	109.4
C5—C6—C12	109.4 (2)	C15—C13—H13	109.4
C11—C6—C12	108.9 (2)	C12—C13—H13	109.4
C5—C6—C7	110.3 (2)	C13—C14—C8	109.3 (3)
C11—C6—C7	108.2 (2)	C13—C14—H14A	109.8
C12—C6—C7	108.5 (2)	C8—C14—H14A	109.8
C8—C7—C6	110.1 (2)	C13—C14—H14B	109.8
C8—C7—H7A	109.6	C8—C14—H14B	109.8
C6—C7—H7A	109.6	H14A—C14—H14B	108.3
C8—C7—H7B	109.6	C10—C15—C13	109.3 (3)
C6—C7—H7B	109.6	C10—C15—H15A	109.8
H7A—C7—H7B	108.2	C13—C15—H15A	109.8
C9—C8—C14	110.1 (3)	C10—C15—H15B	109.8
C9—C8—C7	109.8 (3)	C13—C15—H15B	109.8
C14—C8—C7	109.3 (3)	H15A—C15—H15B	108.3
C9—C8—H8	109.2	C5—N1—C1	124.1 (2)
C14—C8—H8	109.2	C5—N1—H1C	113.9
C7—C8—H8	109.2	C1—N1—H1C	121.8
			
N1—C1—C2—C3	−65.2 (5)	C5—C6—C11—C10	179.5 (2)
C1—C2—C3—C4	−165.0 (5)	C12—C6—C11—C10	58.8 (3)
O1—C5—C6—C11	126.9 (3)	C7—C6—C11—C10	−59.0 (3)
N1—C5—C6—C11	−54.1 (3)	C5—C6—C12—C13	179.0 (2)
O1—C5—C6—C12	−112.6 (3)	C11—C6—C12—C13	−58.9 (3)
N1—C5—C6—C12	66.4 (3)	C7—C6—C12—C13	58.6 (3)
O1—C5—C6—C7	6.6 (4)	C6—C12—C13—C14	−60.3 (3)
N1—C5—C6—C7	−174.4 (2)	C6—C12—C13—C15	60.2 (3)
C5—C6—C7—C8	−178.8 (3)	C15—C13—C14—C8	−58.9 (4)
C11—C6—C7—C8	59.0 (3)	C12—C13—C14—C8	61.0 (4)
C12—C6—C7—C8	−59.0 (3)	C9—C8—C14—C13	59.6 (4)
C6—C7—C8—C9	−60.3 (4)	C7—C8—C14—C13	−61.1 (4)
C6—C7—C8—C14	60.5 (4)	C9—C10—C15—C13	−59.7 (3)
C14—C8—C9—C10	−60.2 (4)	C11—C10—C15—C13	61.0 (3)
C7—C8—C9—C10	60.1 (4)	C14—C13—C15—C10	59.2 (3)
C8—C9—C10—C15	60.3 (3)	C12—C13—C15—C10	−61.0 (3)
C8—C9—C10—C11	−60.1 (4)	O1—C5—N1—C1	2.9 (5)
C9—C10—C11—C6	60.4 (3)	C6—C5—N1—C1	−176.1 (3)
C15—C10—C11—C6	−60.2 (3)	C2—C1—N1—C5	115.9 (4)

### Hydrogen-bond geometry (Å, °)

**Table:**

*D*—H···*A*	*D*—H	H···*A*	*D*···*A*	*D*—H···*A*
N1—H1C···O1^i^	0.99	1.96	2.896 (3)	158

Symmetry codes: (i) *x*, −*y*, *z*−1/2.
